# Low-Cost, Open-Source, High-Precision Pressure Controller for Multi-Channel Microfluidics

**DOI:** 10.3390/bios15030154

**Published:** 2025-03-02

**Authors:** Mart Ernits, Olavi Reinsalu, Andreas Kyritsakis, Veikko Linko, Veronika Zadin

**Affiliations:** 1 Institute of Technology, University of Tartu, Nooruse 1, 50411 Tartu, Estonia; olavi.reinsalu@ut.ee (O.R.); andreas.kyritsakis@ut.ee (A.K.); 2 Department of Bioproducts and Biosystems, Aalto University School of Chemical Engineering, Kemistintie 1, 02150 Espoo, Finland

**Keywords:** pressure controller, microfluidics, Arduino, liposomes

## Abstract

Microfluidics is a technology that manipulates liquids on the scales ranging from microliters to femtoliters. Such low volumes require precise control over pressures that drive their flow into the microfluidic chips. This article describes a custom-built pressure controller for driving microfluidic chips. The pressure controller features piezoelectrically controlled pressure regulation valves. As an open-source system, it offers high customizability and allows users to modify almost every aspect. The cost is roughly a third of what similar, alternative, commercially available piezoelectrically controlled pressure regulators could be purchased for. The measured output pressure values of the device vary less than 0.7% from the device’s reported pressure values when the requested pressure is between −380 and 380 mbar. Importantly, the output pressure the device creates fluctuates only ±0.2 mbar when the pressure is cycled between 10 and 500 mbar. The pressure reading accuracy and stability validation suggest that the device is highly feasible for many advanced (low-pressure) microfluidic applications. Here, we compare the main features of our device to commercially and non-commercially available alternatives and further demonstrate the device’s performance and accessibility in successful microfluidic hydrodynamic focusing (MHF)-based synthesis of large unilamellar vesicles (LUVs).

## 1. Introduction

Microfluidics is a field that manipulates liquids using devices with narrow channels and small chambers on the microscale. It is often utilized to mix or separate different substances efficiently or to produce and modify nanoparticles [1,2]. Other applications include diagnosis devices, such as biochips/lab-on-a-chip/point-of-care devices, cell culture media, nanomaterial synthesis platforms, drug delivery systems, studying blood cell deformability, single-cell analysis, simulating various microbial growth conditions present in hard-to-reach places, nanogel production, and organ-on-chip systems [3,4,5,6,7,8]. For example, the technique has been used to produce rather homogeneous unilamellar lipid vesicles in various sizes [9,10], which are often employed as biomedical cargo carriers for delivery purposes [11]. It is also possible to create liposomes using a hydrodynamic focusing technique in a microfluidic chip [12]. Yet another implementation of microfluidics is the fast and efficient mixing of reagents for a variety of chemical reactions, which is important to minimize the formation of unwanted secondary products [13]. In addition, microfluidic approaches can significantly improve 3D printing, especially in multi-material handling [14].

Microfluidics requires precise control of flow rates as low as 1 μL per minute. Depending on the application, keeping the flow rates constant over an extended period of time can be essential. For example, in the setup presented in Ref. [10], challenges in maintaining constant flow rates limited the yield and homogeneity of produced lipid vesicles. Other examples requiring precise control of flow rates include high-pressure synthesis and precise extraction [15]. The most immediate control over flow rates can be achieved by regulating the pressure at the input and output of the microfluidic device. This is intuitively understandable, as pressure gradients directly govern the movement of liquids in the device’s channels. Commercially available solutions to fulfill this task exist, but they are closed-source, have limited or restricted user control options, and are relatively costly in comparison to open-source solutions [16].

Syringe pumps as flow controllers are a popular choice for microfluidic systems as they are affordable and provide robust and precise flow control [17,18,19]. However, such systems have significant drawbacks, including pressure and flow fluctuations [20,21,22], high dead volumes and sample waste [23], as well as substantial pressure overshooting and steady-state errors [24]. Electronically actuated pressure controllers, such as systems employing solenoid or piezoelectric valves, meet the demands for higher pressure stability applications. While the more robust solenoid valves enable the development of more cost-effective and highly modular systems, the discontinuous operation of such valves still causes noticeable overshooting and oscillation [25]. The continuous pressure control of piezoelectric valves, on the other hand, provides enhanced precision and stability [26]. Therefore, piezoelectric valves are preferred by many leading microfluidic pressure controller manufacturers, such as by Elvesys (Paris, France) in Elveflow product line.

Here, we introduce an alternative, customizable device that provides top-notch pressure stability and can be built significantly more cost-effectively than commercial offerings. It also has a somewhat scalable design, as it is relatively straightforward to add new channels to it by adding valves and mainboards. Our piezoelectric pressure controller (PEPC) is meant to be used in various microfluidics applications. The PEPC takes in positive and negative pressures from a compressor and a vacuum pump and regulates the pressure in its four outlet tubes. It is controlled by a fully open-source controller application that can be customized to add new features, such as interactive experiment sequences or additional calibration steps. The device can also function using just a compressor to regulate only positive pressures.

In this article, we first describe how to construct the device. Then, we characterize its performance by determining the pressure transition times (consisting of the pressure change and stabilization times), and the stability of the outputted pressures. We then compare its performance and main features to similar commercial and non-commercial alternatives. Finally, we show that the PEPC is sufficiently stable for producing large unilamellar vesicles (LUVs) using microfluidic hydrodynamic focusing (MHF). We also provide an intuitive graphical user interface to control the device (available in the Supplementary Materials). The presented system could find uses especially in low-pressure applications where cost-effective, customizable, and stable microfluidic pressure control is needed, ranging from educational settings and small-scale laboratory works to more sizable research projects and manufacturing processes.

## 2. Materials and Methods

### 2.1. Design of the Pressure Controller

The PEPC uses piezoelectric valves from Hoerbiger AB (Zug, Switzerland) to control the pressure in four outlet tubes. The valves internally use a PID control algorithm that has been pre-tuned by the manufacturer. Our PEPC is similar in that regard to the commercially available flow controllers, but since it is open source, the users can customize it to their needs, and it costs only about a third of those devices. In addition, piezoelectric valves are inherently more able to react faster to different inputs than most alternatives. Compared to other solutions, such as solenoid valves, piezoelectric valves can react more quickly and precisely to maintain the requested pressure value in the output [26], for example, in case of changes in input pressure or flow dynamics of the microfluidic channel. Nevertheless, the most desirable feature of our device is to produce as stable output pressures as possible, whereas making actual transition times shorter when changing between different output pressure values is considered less important.

It can be used to control both positive and negative air pressure in the output tubes. It is also possible to use the device when only one positive pressure input is present, while the negative pressure input is just exposed to ambient pressure. It is possible to use either pre-pressurized pressure vessels or active pumps/compressors for the pressure sources. Naturally, the more stable the input pressure, the more stable the output. Mechanical vibrations, such as those stemming from pumping, can be mitigated by using non-rigid and large-volume pipes and buffer tanks in the input.

The device contains an Arduino Uno Rev3 microcontroller board (Arduino, Monza, Italy) that communicates with a special Node.js-based application on a PC via the serial protocol. The protocol is simple, and all calibration and adjustments are performed on the controller’s PC. Namely, the PC sends a command to the device in the form of nX, where n is an integer from 0 to 65535 and X is either A, B, C, or D. For example, the device might send a command to set maximum pressure in the outlet A by sending “65535A”. The device sends a packet of data in JSON format about 5 times per second. The JSON data packet looks like the following: { “A”: 13520, “B”: 13432, “C”: 14112, “D”: 13580, “A_r”: 15000, “B_r”: 0, “C_r”: 0, “D_r”: 0, “multiplier”: 0.000187505722045 }. Here, A, B, C, and D refer to the current readout from the respective outlets, A_r, B_r, C_r, and D_r refer to the values that were requested for these outlets most recently, and the multiplier is the value that the readout values should be multiplied by to yield voltage values. Other strings may also be sent from the device between JSON packets to make debugging easier. Still, these messages can never contain curly braces and, therefore, do not interfere with the basic pattern of the protocol.

**Figure 1:**
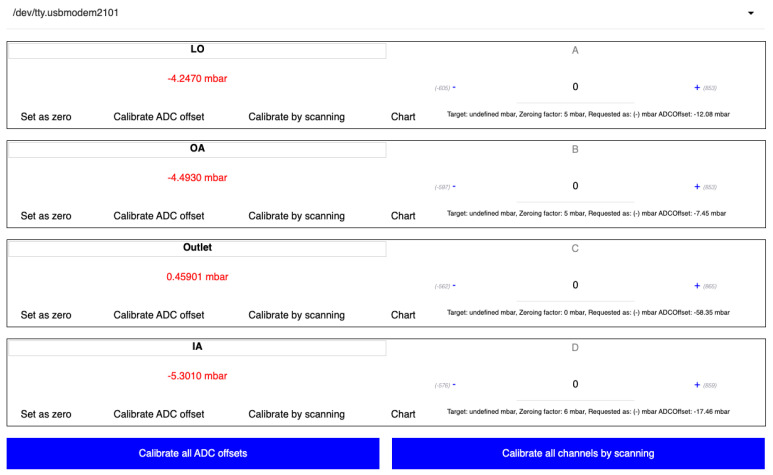
The main view of the controller application. “LO”, “OA”, “Outlet”, and “IA” are values that the user entered to give meaningful names to the channels relating to the experiment being performed. “A”, “B”, “C”, and “D” are physical channel names relating to the physical outlets on the device. ADC denotes analog to digital converter.

The controller application has a browser-based graphical user interface. It is accessed by opening a browser window to http://localhost:7005/#pressure_controller after running the executable file, which can be downloaded from http://doi.org/10.17632/rf5jr78wy7.2 (accessed on 27 February 2025) (inside of “Controller-application.zip”). Figure 1 shows the main view of the application, with a connected device as an example.

If needed, the device can be scaled by adding more channels. The number of channels can be relatively easily increased to eight by adding more valves and another motherboard. However, this would also require modification of the Arduino source code to include the second mainboard by adding a second software-defined I^2^C channel or a small change to the connections on the motherboard to assign a different address to the digital to analog converter (DAC).

The device is enclosed in a plastic toolbox and is, therefore, easy to transport. The pressure inputs and outputs feature quick-connect tube fittings (8 mm and 4 mm tube outer diameter for the inputs and outputs, respectively) and individual valves so that each input can be opened or closed as needed. It requires plugging into a wall socket for power and connecting to a PC via a USB cable. It is made of readily available materials, not 3D printed, but still relatively easy to build.

### 2.2. Build Instructions for the Pressure Controller

**Figure 2:**
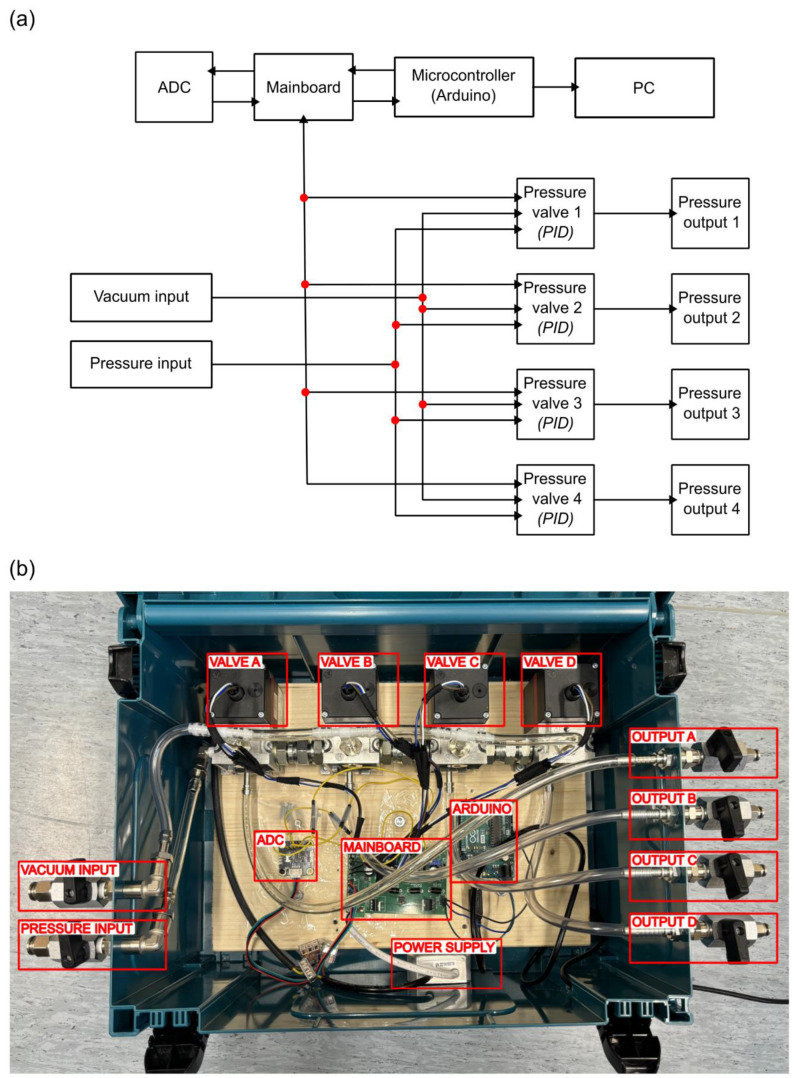
The main components of the device. (**a**) A schematic view showing the main components and their connections. (**b**) A photograph of the fully assembled device with labeled main components (the plastic box size is 395 × 295 × 210 mm). The plumbing-related components are presented in Supplementary Materials Figure S1.

Building the pressure controller starts by assembling the mainboard. JLCPCB, Hong Kong, China (www.jlcpcb.com) (accessed on 27 February 2025) was used as the manufacturer in this case, as it was straightforward to place an order for a printed circuit board together with most of the electrical components attached to it from the software EasyEDA v6.5.34 (JLCPCB) (www.easyeda.com) (accessed on 27 February 2025), which was used to design the board. This step should be carried out first, as it takes time to manufacture the mainboard. Figure 2a shows a schematic view of the main components and connections of the PEPC (including the mainboard). Figure 2b shows an inside view of the device where the main components have been labeled, while a more detailed view of the plumbing components in the device is presented in Supplementary Materials Figure S1. All the mainboard components are listed in Supplementary Materials Table S1. The plumbing-related components have been listed in the bill of materials (Supplementary Materials Table S2, where the labeling corresponds to Figure S1).

Four Hoerbiger Tekno Plus Vakuum proportional pressure control valves should be connected using base plates side by side to connect their pressure inputs. The authors of this paper used G3/8 size fittings for the connections but recommend that future builders of this device use the official connection method from Hoerbiger instead.

Then, the vacuum inputs should be connected using a sufficiently rigid plastic tube and T-junctions. The tube can be made out of polyurethane (PUR) or polyvinyl chloride (PVC) with an inner diameter of 6 mm and an outer diameter of 9 mm. The PUR tubes are much more rigid and should, therefore, be better for pneumatics, but the whole controller could also be built using only the softer PVC tubes.

The next step is finding a sufficiently large container to house the pressure controller system. In this case, a plastic toolbox (MAKITA NR.3, 395 × 295 × 210 mm) was used (Figure 2b).

The pressure control valves should be attached to the inside of the box. In this case, a wooden plate was used as a base to attach the pressure valves and the electronic components to the box. The plate was cut to size to fit it inside the box, and then holes were drilled at the bottom of the box so that the plate could be bolted to it.

It is then necessary to attach the valves to the box and to connect all the wires as per the schematics provided in the Supplementary Materials (https://doi.org/10.17632/rf5jr78wy7.2) (accessed on 27 February 2025). It is important to ensure that the address selector switch on the ADC module is set to 0x49. A 24 V power supply should be connected to the mainboard to provide power to the system. Six valves should be attached to the side of the box—two for the pressure and vacuum inputs and four for the regulated pressure outlets. Then, the tubes leading into and out of the pressure control valves should be connected to the valves leading into and out of the box. The valves should be labeled appropriately.

Connect the Arduino to an external computer using a USB cable. Upload the sketch named “sketch_pressure_regulator.ino” to the microcontroller using the Arduino desktop software that can be downloaded from www.arduino.cc (accessed on 27 February 2025).

### 2.3. Operating Instructions of the Pressure Controller

To use the pressure controller, first connect the pressure and vacuum inputs. The pressure should be between 1.5 and 2.5 bar. The vacuum pressure should be as low/strong as possible, but at least −0.7 bar is recommended. It is possible to use the device with no vacuum pump connected. Still, the reaction time is significantly limited, and the device cannot reliably control output pressures lower than 25 mbar in that mode. In this case, the vacuum input valve should be left open to ensure zero pressure in the input and not closed. Second, plug in the power supply, connect the USB cable from the pressure controller to a PC, and run the controller application on the computer. The application should open a browser window with the URL http://localhost:7005/#pressure_controller. If the window does not appear, then open it manually. Refresh the page if needed until the port of the pressure controller device appears in the device selector dropdown. Select the pressure controller in the device dropdown to connect to it. The software will automatically prompt you to calibrate the device upon first connection. Select “yes” and follow the on-screen instructions to perform calibration.

Automatic calibration involves detecting a “zero ADC offset” value determined by opening all outlets and averaging the reported pressure in each outlet. This average value is then subtracted from all future reported pressure values transmitted by the device. In other words, the controller’s zero value for output pressure is equated with the atmospheric pressure of the surrounding environment. This offset originates from the fact that the voltage reference on the mainboard is not exactly the same as inside the valves. The second step of the automatic calibration procedure requires all the outlets to be closed. In this step, a number of requested pressures across the range of available output pressures from −1000 mbar to 1000 mbar is sampled to obtain a calibration curve. An example curve is shown in Supplementary Materials Figure S2. This curve accounts for the difference between the requested and obtained values observed during the calibration. The software will automatically detect the achievable range of pressure values for each outlet and set the outlet limits accordingly. After calibration, the controls of four pressure outlets will appear.

**Figure 3:**
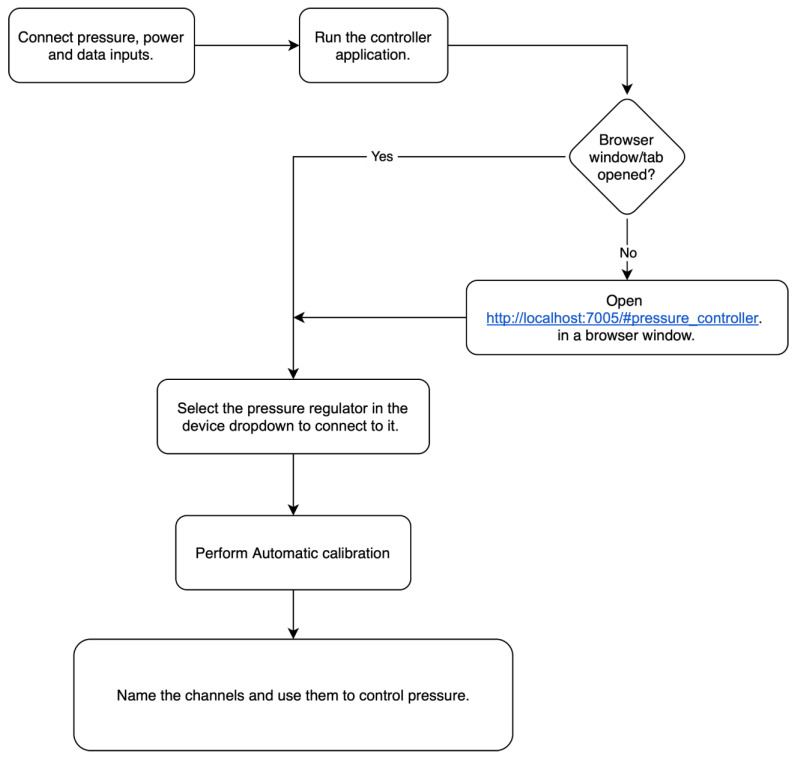
Flowchart of the actions needed to start using the device.

It is possible to write names for each channel in the textbox at the top left of each pressure outlet view. The calibration curve for the associated pressure outlet is displayed when the chart button is clicked. The set-as-zero button is meant to function similarly to the zeroing/tare button on lab scales, adding a constant numerical offset to the outlet so that the value present when pressing the button acts as the zero pressure point. The “+” and “−“ buttons increase and decrease the target pressure by 1 mbar. Typing a target value into the text field between the “+” and “−” buttons is also possible. The target is sent to the pressure controller immediately when typing. There is no need to press enter after typing in a target pressure value. Figure 3 and Table 1 summarize some of this information in a more visual format.

Note that since the presented version of the PEPC does not include a flow-sensing apparatus, the operator will have to take care to avoid any possible backflow by visually monitoring the flow in the chip.

### 2.4. Vesicle Production and Characterization

Large unilamellar vesicles (LUVs) were produced using a microfluidic hydrodynamic focusing (MHF) technique. This approach involved using a microfluidic chip with two inputs and one outlet port for which the flow of the solutions was regulated through the PEPC-controlled pressures. The microfluidic chip was made of polydimethylsiloxane (PDMS) and bonded to glass in the same way as in Ref. [10]. One input was a solution containing 3.9 mM of dipalmitoylphosphatidylcholine (DPPC), 0.1 mM of dipalmitoylphosphatidylglycerol (DPPG), and 1 mM of cholesterol in ethanol. The second input channel was fed with Milli-Q water. The chip contains one intersection where the lipid-ethanol solution is jetted into a stream of water. The pressures applied to the two input ports and one outlet port of the chip were varied until a stable jet was obtained. With this approach, LUVs with a diameter of ~100 nm are spontaneously formed as the ethanol mixes with water. This approach is described in more detail in Refs. [27,28].

The produced LUVs were visualized using negative staining transmission electron microscopy (TEM). A 5 μL sample of LUV suspension was applied onto a formvar–carbon-coated copper grid, incubated for 5 min to associate vesicles with the grid, and stained with 2% aqueous uranyl acetate solution for 2 min. After washing with a drop of deionized water and air drying, the samples were analyzed with a Tecnai G2 Spirit BioTwin (FEI, Hillsboro, OR, USA) transmission electron microscope at a 120 kV accelerating voltage, and images were captured using an Orius SC1000 camera (Gatan Inc., Pleasanton, CA, USA).

In addition to TEM characterization, dynamic light scattering (DLS) was used to analyze the LUV suspensions. Measurements were performed using a Zetasizer Ultra (Malvern Panalytical, Spectris PLC, London, UK). The sample was diluted 50 times in Milli-Q water, and 1 mL of the suspension was analyzed using a measurement cuvette DTS0012. The refractive index was set to 1.45, absorption to 0.001, and the dispersant was set as water in the ZS XPLORER software v3.31 (Malvern Panalytical). Three replicas were analyzed using particle size and concentration measurement to obtain a size distribution and a polydispersity index.

## 3. Results and Discussion

### 3.1. Validation of the Pressure Reading Accuracy Using a Manometer

The accuracy of the internally reported pressure readings of the device was determined by comparing the reported values with those measured using a calibrated differential manometer (Kane 3500-5, Kane International Limited, Welwyn Garden City, UK). The output pressure was set to a particular requested value, and then five readings were taken simultaneously from the device and the manometer. The differences between the internal and manometer readings (i.e., the reporting errors) were averaged and presented in Table 2, along with the requested target pressures. The reporting error was determined by subtracting the pressure value that was reported by the device from the corresponding reading obtained using the manometer. The ± values represent standard deviations (*n* = 5). A constant was added to all the values to set the reporting error to 0.00 mbar for the requested value of 0 mbar. The software can consider this constant by using the “set as zero” button in the interface. The available measurement range of the manometer limited this test to be performed between −380 and 380 mbar. However, this is sufficient to provide a reasonable overview of the reading accuracy and to reveal any possible trend in the errors in this pressure range that is relevant for many low-pressure microfluidic applications such as liposome production [10], organ-on-chip methods [29], microfluidic chemical testing [30], on-chip pressure sensing [31], cavitation research [32], microfluidic mixing [33], and cell culture and liquid dosing applications [34,35]. In this range of pressures, the average relative reporting error of the PEPC remained below 0.7%, as seen in Table 2. The highest reporting error (0.67 ± 0.01%) was observed in the case of −380 mbar of the requested pressure. In general, the error looks to increase with the increasing absolute value of the requested pressure, and it is more pronounced in the case of negative pressures. It can be speculated that the relatively weak vacuum pump, in this case, limits the pressure stability at the more extreme negative pressure values.

### 3.2. Characterization of the Pressure Controller’s Performance

To test the performance of the PEPC, an experimental setup was installed where the positive input pressure was set to 2 bar from a TC-30T compressor (Wenzhou Hanfong Machinery Co., Wenzhou, China), and the negative pressure input of the PEPC was connected to a LABOPORT^®^ N 820.3 FT.18 vacuum pump (KNF Neuberger GmbH, Freiburg im Breisgau, Germany). The outlets were opened and connected to a 33 cm long tube with an inner diameter of 0.5 mm.

**Figure 4:**
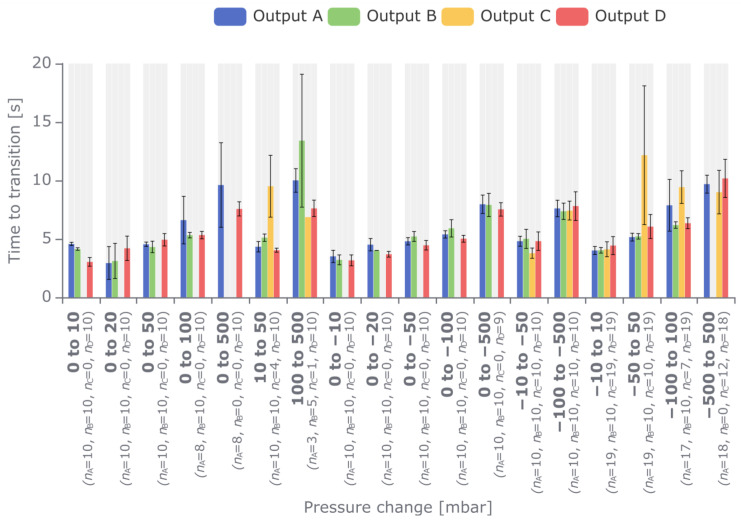
Average time in seconds taken to transition between various output values. The different color bars represent the average transition times for each output (Output A, blue; Output B, green; Output C, yellow; Output D, red). The error bars show the standard deviations of these averages. The *n* values under each bar indicate the number of samples used for the given bar. In some cases, a bar is missing, or the *n* value is less than 10. This means the signal failed to transition within 25 s, and the result was ignored.

An experiment was designed where the pressure was cycled between various pressure values by setting the target pressure value and waiting for the pressure transition to complete. A total of 18 different pressure intervals were measured in a range between −500 and 500 mbar for each outlet (Figure 4), allowing for the assessment of the time required to achieve the needed pressures from various starting points. Each pressure interval was scanned at least 10 times. The pressure was observed by recording the self-reported output pressure values of the pressure regulation valves. The controller application was programmed to perform the scan, wait for signal transitioning, and then record the signals from the start of transitioning until 5 s after the signal was successfully transitioned to a new pressure level. A signal is considered transitioned if, when considering a 5 s stretch of the signal as a sliding window, the average values recorded during the first and the third second are within ±0.25 mbar of the average value obtained during the fifth second. The first datapoint of the mentioned 5 s signal is then considered the point at which the signal has completed transitioning. The transition time of PEPC appears to be at least 3 s and, in some cases, can be as large as 10 s when the pressure change is large. Interestingly, it takes less time to move from 0 mbar to 20 mbar than it takes to move from 0 mbar to 10 mbar. The transition time can vary significantly between attempts, sometimes even as much as 6 s. This is likely due to the particulars of the PID algorithm governing the pressure valves, which is path-dependent. Notably, the variability of the transition time is higher when moving from a lower pressure towards higher pressure values. This correlates with the asymmetry of the pressure inputs, where the positive pressure input at 2 bar is approximately 2.9 times as strong as the vacuum one at −0.7 bar. Nevertheless, such long transition times are still sufficient for many types of microfluidic experiments, where very short reaction times are not necessarily required.

**Figure 5:**
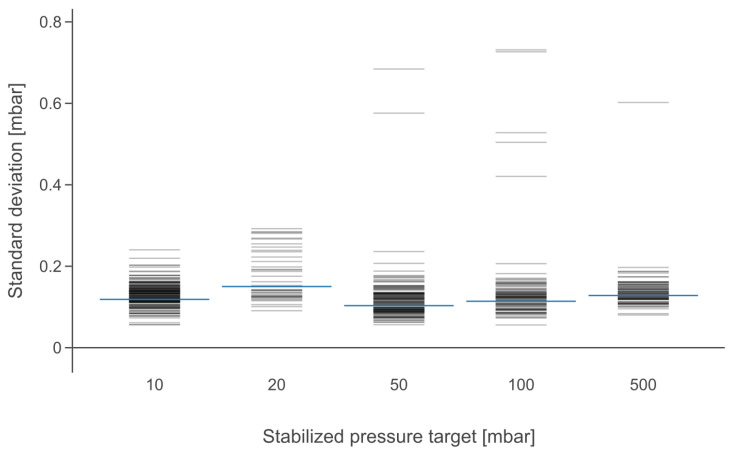
Standard deviations of sample signal points at different target pressures (stabilized, 5 s samples). The dataset excludes transitioned signals that have not yet stabilized by filtering out signals where the standard deviation is over 0.75 mbar. The blue lines indicate the average values and the light gray ones represent individual values.

To characterize the pressure stability of the PEPC’s output, the standard deviations of the stabilized 5 s long output pressure signals at different pressure target values (from 10 to 500 mbar) were calculated. These deviations are presented in Figure 5. A signal was considered to be stabilized if transitioning after a target pressure change was completed and the standard deviation of the pressure value was lower than 0.75 mbar. It was possible to find instances where the pressure in an outlet had finished transitioning to a new level. However, it was still oscillating for a while before stabilizing, and these instances were found to have standard deviations over 0.75 mbar. The standard deviation values above 0.4 mbar (in cases of 50, 100 and 500 mbar) could be called outliers when looking at the data after filtering out the non-stabilized samples. Still, they appear to belong to the stabilized group when looking at the unfiltered data (see Supplementary Materials Figure S3). The pressure output has an average standard deviation of <0.15 mbar in cases where the pressure has stabilized (blue lines in Figure 5). The obtained level of stability is more than sufficient for microfluidic applications such as liposome production using hydrodynamic focusing. Notably, this stability was measured with the relatively noisy pressure sources of a compressor and a vacuum pump.

### 3.3. Pressure Controller’s Performance Compared to Alternatives

We have validated and characterized our PEPC based on its most relevant features. To place these characteristics into perspective, we have compared the PEPC’s performance to three commercial and non-commercial alternatives and summarized the results in Table 3. The commercial devices compared are the Elveflow OB1^TM^ Mk4 (specifically the version with a pressure range of −900–1000 mbar) [36], Fluigent MFCS^TM^ [37], and Biophysical Tools P^2^CS^TM^ [38]. The transition time described above is not directly comparable to the available response and settling times for the listed alternatives. For this reason, the values shown in Table 3 have been specifically calculated to match the testing criteria described for the Elveflow OB1^TM^ [36]. Based on the data shown in Figure 4, the calculated response time of the PEPC represents the average time to reach 5% of the setting point, whereas the settling time represents the average time to reach 95% of the setting point (the criteria used for the commercial devices). The settling time of the PEPC is much longer than the settling times reported for the alternatives. This can partly be explained by presumably larger output tubes in the PEPC than those used in the alternatives and by using electronic low-pass filters on the mainboard with a low cutoff frequency. Since the main focus of the PEPC was to develop a pressure regulator that would provide stable input for a microfluidic experiment, optimizing the reaction time was not a priority. Thus, while it may have a limited reaction capability, the PEPC shows high-quality pressure stability that is comparable to the leading pressure controller offerings in the market.

The outstanding pressure stability of the PEPC is even more pronounced when compared to open-source and non-commercial pressure control devices. For example, Sanchez and Chang have presented a solenoid valve-based controller system [39] and compared it to other solenoid valve-based [40] and piezoelectric pressure controllers [16] that are only able to regulate positive pressures. All of these devices exhibit at least two times as high pressure fluctuations as the PEPC (Table 3). Therefore, our PEPC, with enhanced pressure stability, could find uses in microfluidic applications that demand higher-grade pressure stability, such as the generation of monodisperse droplets [41] and liposomes [10], as well as microfluidic single-cell manipulation and analysis methods [42].

On the other hand, the accuracy and reaction time characteristics of the PEPC are limited compared to these three devices. These devices feature at least two times as high accuracy as the PEPC, and if stricter requirements for quicker pressure changes are necessary, then the alternatives could serve as better choices (however, the way the reaction times of these three devices have been characterized does not allow a direct comparison with this work). As with open-source solutions, the PEPC and the alternatives are more affordable than commercial alternatives, but they require the availability of resources and special skills to assemble them.

### 3.4. Vesicle Production Using the Pressure Controller in Microfluidic Hydrodynamic Focusing

**Figure 6:**
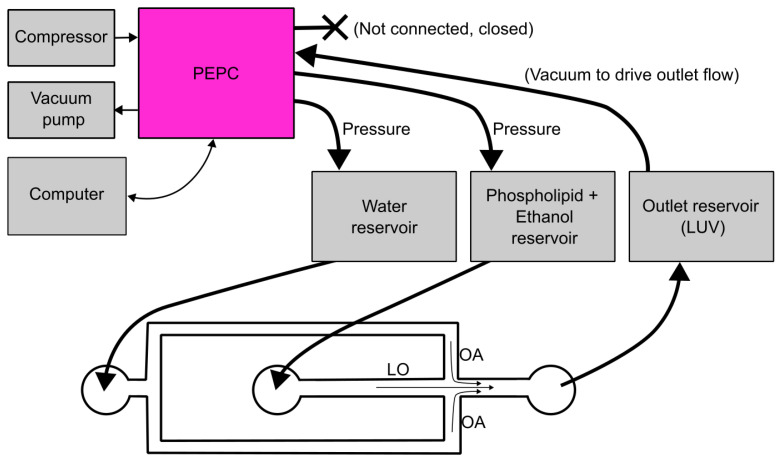
Schematics of the experimental setup for microfluidic production of LUVs using MHF. The system uses both positive and negative input pressures. LO and OA correspond to the same custom names assigned to outlets in Figure 1.

Besides validating and testing the device’s performance by measuring the output pressures using a precision manometer and defining the stability and response times, its applicability was also demonstrated in MHF-based production of large unilamellar vesicles (LUVs). The schematic of the production setup is shown in Figure 6. Two pumps were connected to provide positive and negative pressure to the pressure controller, and a computer was used to control the pressures outputted by the PEPC. Two outputs were set to positive pressure values and connected to input solution reservoirs to push liquids into the chip. One outlet was set to negative pressure to guide the formed LUVs from the chip into a collection reservoir. One pressure outlet was left closed and not connected. In the chip, two liquids were mixed by jetting the liquid that contained phospholipids into water.

**Figure 7:**
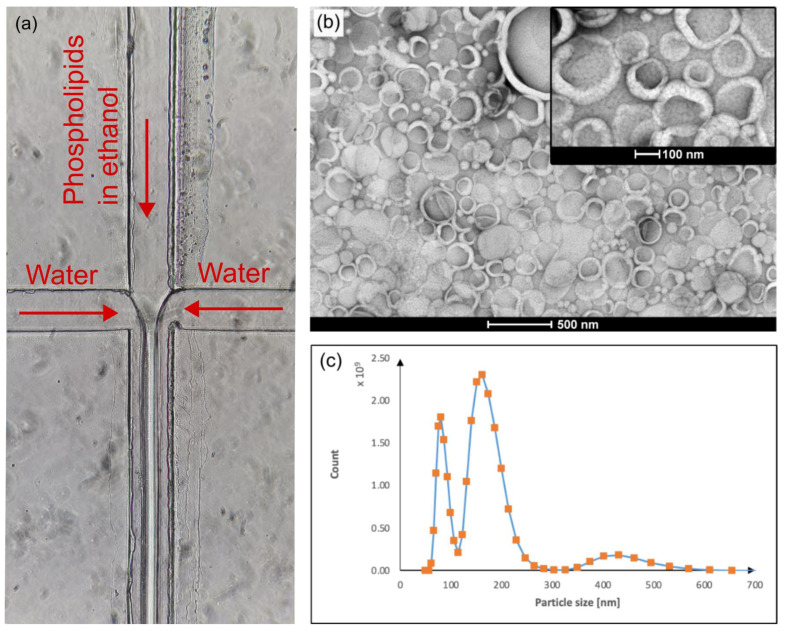
Microfluidic production of LUVs using MHF. (**a**) Image of the chip during the production process. Phospholipids dissolved in ethanol are jetted into a stream of water. The jet remained stable for the duration of the entire experiment. The visible irregular area around the channels originates from the roughness of unbonded PDMS and does not affect the chip’s functionality and experiment in any significant way. (**b**) Negative-stain TEM image of the produced LUVs. (**c**) The size distribution of the produced LUVs, as determined by DLS.

Figure 7a shows the view of the main junction of a microfluidic chip used to produce LUVs using the MHF method. The jet, which can be seen in the image, remained stable for the duration of the experiment, which took about 10 min. TEM imaging revealed that LUVs of the expected size of about 100–200 nm were successfully produced in the experiment, as seen in Figure 7b. Additional DLS analysis (Figure 7c) showed a particle size distribution with two peaks around ~80 nm and ~180 nm, matching the diameters of LUVs seen in the TEM image. In addition, a less pronounced peak between 400 and 500 nm was observed, which also agrees well with the TEM observation. The polydispersity index in the DLS experiment was 0.45.

## 4. Conclusions

In conclusion, we have designed a programmable microfluidic pressure controller and reported, in detail, a way to construct it. We have validated its accuracy and proven that it can reliably provide stable pressure levels, which are often requested in microfluidics. Our PEPC exhibits similar stability and accuracy compared to commercial and non-commercial devices. The reaction times of our PEPC device are longer than the ones reported for the alternatives. However, in this work, there was no imminent need to improve the reaction times beyond what they currently are. To further emphasize the feasibility of the device in real-life applications, we have also demonstrated that the PEPC can be harnessed in the synthesis of liposomes/vesicles using a microfluidic setup. Based on the cost of materials, this device is significantly more cost-effective than similar alternative commercially available offerings while still including piezoelectric output pressure control. While the device does not offer direct flow control for microfluidics—as it does not include a flow sensor that could provide the necessary feedback for such functionality—this feature could be relatively easily added because it is open source. As it is, our modifiable controller is suitable for (low-pressure) microfluidic experimenting where the exact flow rate is not as important as the topological movement of liquids within a microfluidic chip. As we have also supplemented our device with a user-friendly open-source interface, we believe this work will serve as a beneficial guide for developing custom microfluidic setups at low cost and ease for various applications.

## Figures and Tables

**Table 1:** Input parameters to use the device.

Input Parameter	Value
Positive input pressure	1.5 to 2.5 bar
Negative input pressure	0 to −1 bar; −0.7 or lower is recommended
Power source voltage	220 V AC

**Table 2:** Difference (reporting error) between the device’s reported value and the output pressure value measured with a calibrated manometer.

Requested Value (mbar)	Average Reporting Error (mbar)	Average Relative Reporting Error (%)
−380	2.55 ± 0.10	0.67 ± 0.03
−200	0.62 ± 0.16	0.31 ± 0.08
−50	0.10 ± 0.13	0.20 ± 0.26
0	0.00 ± 0.08	N/A
50	−0.09 ± 0.13	−0.19 ± 0.25
200	0.47 ± 0.17	0.23 ± 0.08
380	1.48 ± 0.11	0.39 ± 0.03

**Table 3:** Comparison of the PEPC performance/features with various commercial alternatives (FS = full scale).

	PEPC	Elveflow OB1^TM^	Fluigent MFCS^TM^	Biophysical Tools P^2^CS^TM^	Ref. [39]	Ref. [40]	Ref. [16]
Pressure range [mbar]	−1000 to 1000	−900 to 1000	0 to 1000	−1000 to 1000	0 to 690	0 to 2200	0 to 2000
Stability	0.006% FS	0.005% FS	<0.1%	0.007% FS	0.01% FS	0.3% FS	0.02% FS
Response time [ms]	69	10	10	4	-	-	-
Settling time [ms]	2100	50	-	5 or 17	-	-	-
Min increment (step size)	0.05% FS * 0.0015% FS (theoretical)	0.0064% FS	0.03% FS	0.006% FS	-	-	-
Accuracy	<0.7% **	2.5%	0.25% FS	0.5% FS	0.16% FS	0.37% FS	0.09% FS

* Arbitrarily limited in software. ** In the range of −380–380 mbar.

## Data Availability

The original contributions presented in this study are included in the article/Supplementary Materials. Further inquiries can be directed to the corresponding authors.

## Supplementary Materials

The following supporting information can be downloaded at: https://www.mdpi.com/article/10.3390/bios15030154/s1, Figure S1: inside view of the device with labeled components; Table S1: electronics components; Table S2: plumbing-related components; Figure S2: example of a calibration curve of the device; Figure S3: Figure 5 without filtering out the signals that had not yet stabilized. The other supplementary materials, including the schematics and software source code, are available at DOI: 10.17632/rf5jr78wy7.2.
