# Supplementary material for: Low-Cost, Open-Source, High-Precision Pressure Controller for Multi-Channel Microfluidics
Source: Biosensors (Basel). 2025 Mar 2;15(3):154. doi: 10.3390/bios15030154 (PMC11940448; doi:10.3390/bios15030154)
Supplement: Supplementary file 1 [file biosensors-15-00154-s001.zip › biosensors-3481491-supplementary.pdf]

Supplementary Materials for

Low-Cost, Open-Source, High-Precision Pressure Controller for Multi-Channel Microfluidics

Mart Ernits[1,*], Olavi Reinsalu[1], Andreas Kyritsakis[1], Veikko Linko[1,2,*], Veronika Zadin[1,*]

[1] Institute of Technology, University of Tartu, Nooruse 1, 50411 Tartu, Estonia
[2] Department of Bioproducts and Biosystems, Aalto University School of Chemical Engineering, Kemistintie 1, 02150 Espoo, Finland

* Correspondence: mart.ernits@ut.ee (M.E.); veikko.pentti.linko@ut.ee (V.L.); veronika.zadin@ut.ee (V.Z.)

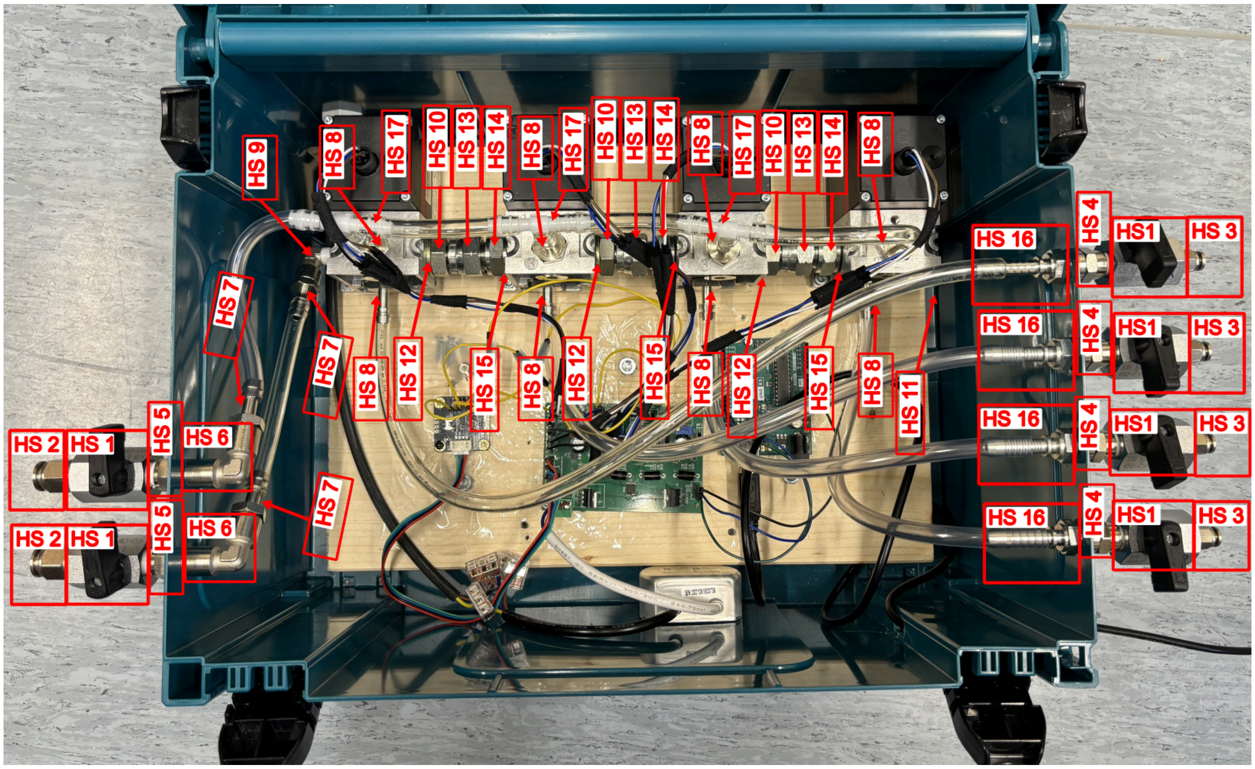

Figure S1. Inside view of the device. Plumbing components/fittings other than tubes are labeled as in the bill of materials (Table S2).

Table S1. Electronics components.

Mainboard components								
**Name**	**Designator**	**Footprint**	**Qty**	**Part nr.**	**Manufacturer**	**Supplier**	**Supplier Part nr.**	**Price**
330n	C1,C7	C0603	2	CGA3E1X7R1C334KT0Y0N	TDK	LCSC	C531425	0.026
100n	C2,C6	C0603	2	CC0603MRY5V8BB104	YAGEO(国巨)	LCSC	C519502	0.004
150u	C8,C9,C10,C11	150UF-35V-NICHICON-CAPACITOR	4					
0.1u	C12,C15,C17,C19	C0603	4	CC0603MRY5V8BB104	YAGEO(国巨)	LCSC	C519502	0.004
1u	C13,C14,C16,C18	C0603	4	CC0603KRX5R8BB105	YAGEO(国巨)	LCSC	C14664	0.005
1.5KE18CA-T	D2,D3,D4,D5	DO-201_BD5.0-L9.0-P13.00-D1.0-BI	4	1.5KE18CA	BORN(伯恩半导体)	LCSC	C600796	0.154
HDR-M-2.54_1X2	H1,H2,H3,H4	HDR-M-2.54_1X2	4			LCSC	C49661	0.036
Pressure valve connector	J1,J2,J3,J4	HDR-M-2.54_2X2	4			LCSC	C66690	0.021
24V input	P2	CONN-TH_2P-P5.00	1	MX126R-5.0-GN01-2P-Cu-S-A	MAX(迈旭)	LCSC	C5188436	0.064
5.2k	R4,R5,R6,R7	R0603	4	0603WAD5231T5E	UNI-ROYAL(厚声)	LCSC	C51223	0.014
10k	R8,R11,R13,R15	R0603	4	AR03BTBX1001	Viking(光颉)	LCSC	C3013536	0.105
5.6k	R9,R10,R12,R14	R0603	4	0603WAF5601T5E	UNI-ROYAL(厚声)	LCSC	C23189	0.001
100K	RP3,RP4,RP5,RP6	RES-ADJ-TH_3386P	4	3386P-1-103	BOCHEN(博晨)	LCSC	C118953	0.2
5K	RP7,RP8,RP9,RP10	RES-ADJ-TH_3296W	4	3296W-1-502LF	BOURNS	LCSC	C60620	0.712

L7812CV-DG	U2	TO-220-3_L10.0-W4.5-P2.54-L	1	L7812CV-DG	ST(意法半导体)	LCSC	C2914	0.177
LR7805-J	U6	TO-220_L10.2-W4.6-P2.54_LR7805	1	LR7805	LRC(乐山无线电)	LCSC	C2846986	0.243
1N5819/SOD-123	U7,U8,U11,U12,U14,U15,U17,U18	SOD-123_L2.7-W1.7-LS3.8-RD	8	1N5819HW	TECH PUBLIC(台舟)	LCSC	C2905649	0.02
LM358P	U9,U10,U13,U16	TSSOP-8_L4.4-W3.0-P0.65-LS6.4-BL	4	LM358PWR	TI(德州仪器)	LCSC	C7951	0.137
DAC8574IPW	U5	16-TSSOP	1			Digikey	DAC8574IPW	19.46

Other electronics

Name	Designator	Footprint	Qty	Part nr.	Manufacturer	Supplier	Supplier Part nr.	Price
DFR0553			1	DFR0553		Digikey	DFR0553	$9.90
Arduino Uno Rev3			1			Oomipood		32 EUR
24VDC power supply			1	PB032624		Oomipood		16EUR

Table S2. Plumbing related components.

Label	Code	Description	Quantity	Unit price (EUR)
HS 1	80950202	Ball valve Mini 1/8" inner thread/outer thread	6	7
HS 2	PF20100802	Pneumatic joint A1 Ø8-1/8" outer thread	2	1.68
HS 3	PF20100402	Pneumatic joint A1 Ø4-1/8" outer thread	4	1.47
HS 4	PF50200202	Sleeve joint B20 G1/8 inner thread	4	0.89
HS 5	PF50410202	Converter joint B41 R1/8 outer thread - G1/8 inner thread	2	0.89
HS 6	PF50700002	Knee joint B70 G1/8 inner thread	2	1.67
HS 7	PF51000702	Tube barb connector B100 7-G1/8 OR	3	1.17
HS 8	PF51000704	Tube barb connector B100 7-G1/4 OR	8	1.67
HS 9	PF50710002	Knee joint B71 G1/8 inner thread - R1/8 outer thread	1	1.48
HS 10	PF50300602	Converter joint B30 G3/8 outer thread - G1/8 inner thread	1	1.25
HS 11	PF50500006	Cap B50 G3/8 outer thread	1	1.36
HS 12	56010106	Gasket Usitr R 3/8"	3	0.35
HS 13	70020606	Nipple G 3/8" x G 3/8" outer thread	3	1.67
HS 14	70080606	Nipple with nut G 3/8" x G 3/8" outer thread-inner thread	3	4.02
HS 15	5K00170140025	OR 14 x 2.5 NBR70	3	0.59
HS 16	42020204	Nozzle G 1/8" outer thread 1/4"	4	2.87
HS 17	50473004	Plastic T-junction 6 mm	3	1.73
HS 18	14290609B	Tube PUR oil/gasoline 6 x 9 mm	1 m	3.83
HS 19	14290609	PVC tube 6 x 9 mm	1 m	1.04

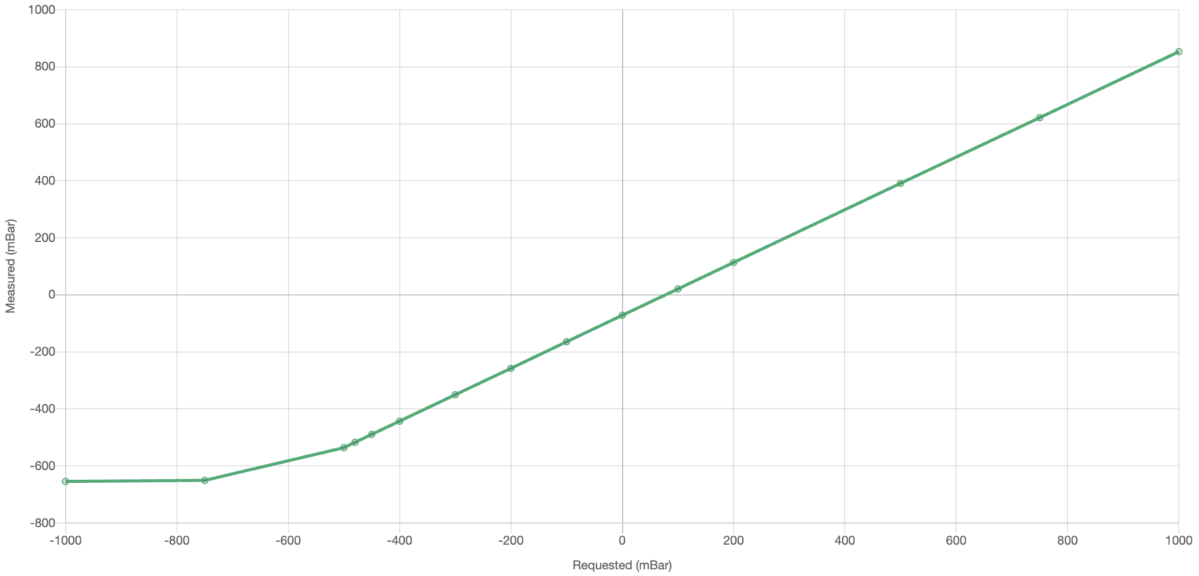

Figure S2. Example of a calibration curve from the automatic calibration procedure of the device.

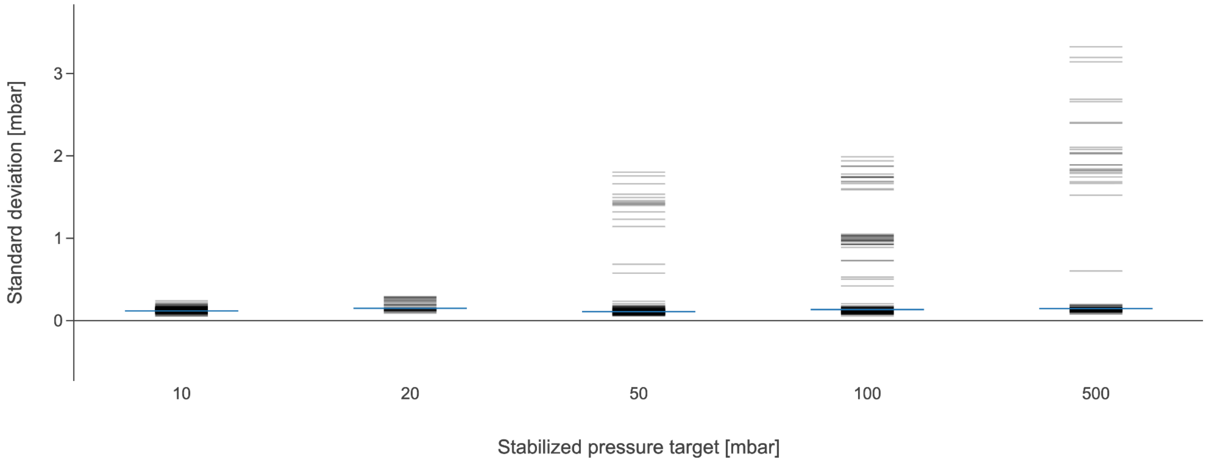

Figure S3. Main article Figure 5 without filtering out the signals that had not yet stabilized.
